# Digital health determinants & divide in the Arab world: A cross-sectional study

**DOI:** 10.1371/journal.pone.0338299

**Published:** 2025-12-31

**Authors:** Radwan Qasrawi, Reema Tayyem, Suliman Thwib, Ghada Issa, Malak Amro, Razan AbuGhoush, Haleama Al Sabbah, Khlood Bookari, Noor Alawadhi, Sabika Allehdan, Hana Trigui, Elie Sokhn, Yousef Khader, Eman Badran, Iman Kamel, Atiyeh Abdallah, Mohamed Jemaà, Emmanuel Musa, Jude Dzevela Kong

**Affiliations:** 1 Department of Computer Science, Al-Quds University, Jerusalem, Palestine; 2 Department of Computer Engineering, Istinye University, Istanbul, Turkey; 3 Department of Human Nutrition, College of Health Sciences, Qatar University (QU)-Health, Qatar University, Doha, Qatar; 4 The Center of Technology and Innovation, Al-Quds University, Jerusalem, Palestine; 5 Department of Public Health, College of Health Sciences, Abu Dhabi University, United Arab Emirates, Dubai,; 6 Department of Clinical Nutrition, Faculty of Applied Medical Sciences, Taibah University, Medina, Saudi Arabia; 7 School of Education, Kuwait University, Kuwait, Kuwait; 8 Department of Biology, College of Science, University of Bahrain, Zallaq, Kingdom of Bahrain; 9 Laboratoire de Microbiologie Moléculaire, Vaccinologie et Développement Biotechnologique, Institut Pasteur de Tunis, the University of Tunis El Manar, Tunis, Tunisia; 10 Molecular Testing Laboratory, Medical Laboratory Department, Faculty of Health Sciences, Beirut Arab University, Beirut, Lebanon; 11 Department of Public Health, Faculty of Medicine, Jordan University of Science and Technology, Irbid, Jordan; 12 Pediatric Department, Faculty of Medicine, Neonatal-Perinatal Division, University of Jordan, Amman, Jordan; 13 National Research Centre, Cairo, Egypt; 14 Department of Biomedical Sciences, College of Health Sciences, QU Health Sector, Qatar University, Doha, Qatar; 15 Human Genetics Laboratory, Faculty of Medicine of Tunis, Tunis El Manar University, Tunis, Tunisia; 16 Neurophysiology, Cellular Physiopathology and Valorisation of Biomolecules Laboratory, Faculty of Sciences of Tunis, Tunis El Manar University, Tunis, Tunisia; 17 Department of Biology, Faculty of Sciences of Tunis, Tunis El Manar University, Tunis, Tunisia; 18 AI4PEP York University, Toronto, Canada; 19 Department of Mathematics and Statistics, Faculty of Science, York University, North York, Ontario, Canada; Sultan Qaboos University College of Medicine and Health Science, OMAN

## Abstract

**Background:**

Digital determinants of health include key technological factors such as internet access, digital literacy, and the quality of online health information. These elements critically influence health outcomes and behaviors.

**Methods:**

This study examined the impact of digital health determinants on health improvement across ten Arab countries: Bahrain, Palestine, Lebanon, Jordan, Kuwait, the United Arab Emirates, Saudi Arabia, Egypt, Morocco, and Tunisia. The study analyzed a dataset of 12,522 samples after implementing SMOTE-ENN to balance underrepresented demographics, capturing data on digital literacy, internet access, and the impact of online health information on personal health.

**Results:**

Results showed that 93.9% of participants reported having internet access, yet 71.4% did not receive formal education on internet usage. Morocco, Tunisia, and Jordan reported the highest percentages of individuals without such education. Regarding health impacts, 32.9% of participants reported significant personal health improvements linked to digital determinants. Egypt, Lebanon, and Saudi Arabia had higher rates of positive health impacts, while Morocco, Jordan, and Bahrain reported the lowest health improvements. Higher digital literacy and reliable internet access were positively associated with better health outcomes across all countries, whereas specific sociodemographic and digital factors varied: younger age and urban residence were linked to greater benefit in the Gulf; education level and healthcare access were especially influential in North Africa; and in the Levant, digital literacy and use of trusted health sources showed strong impact. These findings show both shared and region-specific drivers of digital health benefits.

**Conclusion:**

Improving health outcomes requires diversification: foundational education on internet usage must be combined with broader digital literacy initiatives, efforts to build and maintain trust in credible online health platforms, and strategies that actively foster patient engagement through interactive digital tools. Policies should also ensure reliable internet infrastructure and tailor interventions to regional and sociodemographic contexts to improve overall health outcomes.

## 1. Introduction

Digital health has a significant positive impact on health systems and is actively transforming healthcare delivery frameworks [1]. However, as healthcare relies more on digital tools and technologies, the digital divide is widening and threatens to exacerbate access disparities [2]. Although this issue is understudied, it is particularly pronounced in the Arab Middle East and North Africa region due to its diverse socio-economic conditions and differing levels of technological infrastructure [3,4]. The digital divide refers to the persistent gap in access to digital technologies such as the internet, computers, and smartphones across various populations and regions. This divide extends beyond simply owning devices and includes factors like affordability, internet speed, digital literacy, and technological skills [5]. Populations that experience poorer health outcomes often continue to face these disadvantages despite technological advancements, as the digital divide can limit their ability to benefit from improvements in healthcare technology and access to digital health resources [6].

Digital determinants of health, including internet access, availability of devices, digital literacy, algorithmic transparency, and exposure to misinformation, exert both direct and indirect influences on health outcomes [7]. These elements affect not only individual engagement with telemedicine, mobile health applications, and online health information but also contribute to broader systemic inequities. For example, the absence of reliable broadband connectivity or adequate digital skills can limit access to remote care and timely health information, thereby exacerbating disparities in chronic disease management, preventive screenings, and health behavior interventions [8]. In contrast, strong digital infrastructure and proficient digital literacy can empower patients by facilitating the monitoring of vital signs, supporting adherence to treatment regimens, and reducing hospital admissions, as demonstrated in mobile health–based improvements in diabetes management [9]. Digital determinants intersect with traditional social determinants of health, such as income, education, and geographic location, forming a complex causal network. This can enhance health outcomes among well-resourced populations while simultaneously widening the health divide for vulnerable groups, ultimately shaping both the equity and effectiveness of health outcomes in the digital age.

Information and communication technologies (ICTs) enhance healthcare by scaling information processing, streamlining administrative tasks, and facilitating access via virtual assistance [10,11]. Proper governance of digital transformations is critical to preventing technology-driven health inequities since weak governance can lead to uneven impacts that worsen health inequalities [12–14]. Digital health presents a contradiction since innovations that improve healthcare delivery for underserved populations also risk excluding these groups due to socio-demographic barriers, especially in areas where inequalities are most pronounced [15,16].

Among the globally recognized determinants of digital health, internet access is most tangible as it mediates health information dissemination, telehealth services, and patient education. Increased internet penetration thereby improves health outcomes through better access to health information and services [4,17,18]. However, significant imbalances in internet access within the Arab MENA region contribute to a digital divide that challenges equitable health outcomes [19,20].

Digital literacy, which is defined as the ability to effectively use digital tools and technologies, is crucial for leveraging the benefits of internet access in healthcare [7,20,21]. Higher levels of digital literacy enable individuals to access, understand, and apply health information, resulting in better health management and outcomes [11,22]. As the cornerstone of the digital divide, digital literacy has thus emerged as a new health determinant [7]. Influenced by factors such as age, education level, and socioeconomic status, digital literacy in Arab MENA is widely varied. Efforts to improve digital literacy must therefore address these disparities to ensure all populations can benefit from relevant digital health innovations [21].

This study aims to assess the impact of digital determinants on health in the Arab MENA region and their relationship to the drivers of the digital divide. This paper explores how emerging digital advancements can improve health outcomes and reduce disparities. It ultimately aims to inform policy recommendations that foster equitable access to digital health resources.

## 2. Methods

### 2.1. Study design and sampling

A cross-sectional survey design was employed to examine the impact of digital determinants on individual health across ten Arab countries: Palestine, Lebanon, Jordan, Kuwait, the United Arab Emirates, Saudi Arabia, Bahrain, Egypt, Morocco, and Tunisia. Participants were selected using a convenience sampling method, with recruitment conducted through social media platforms, online discussion forums, university mailing lists, and community centers to maximize diversity and geographic coverage.

Calculating the required sample size was a critical step in ensuring methodological accuracy. The questionnaire consisted of 50 items, excluding socio-demographic variables. Following widely accepted methodological guidelines suggesting a minimum five to ten respondents per questionnaire item [23], the study aimed for a target of 500 participants per country. Given the challenges of multi-country recruitment and the need for feasible yet valid subgroup analyses, a conservative target of 300–400 respondents per country was established, corresponding to an overall minimum of approximately 3,000–4,000 participants. This target allowed for sufficiently narrow 95% confidence intervals (±5–6%), enabled meaningful stratification by gender and age groups, and ensured statistical power and comparability across countries. In practice, the study substantially exceeded these thresholds by collecting 6,280 valid responses after data cleaning, thereby reinforcing the reliability, robustness, and generalizability of the findings.

To enhance credibility and representativeness, clear inclusion and exclusion criteria were applied. Eligible participants were adults aged 18–64 years, residing in one of the ten target countries, able to read and respond in Arabic or English, and willing to provide informed consent. Responses from individuals under 18 years old, those living outside the target countries, incomplete or duplicate submissions, and automated or spam entries detected through quality checks were excluded from the analysis.

To ensure diversity, particular attention was paid to gender balance and to achieving adequate representation across three age groups: 18–34, 35–44, and 45–64 years. Recruitment quotas were applied where necessary to reduce the risk of overrepresentation from certain groups, such as younger individuals more active on social media platforms, and to secure a balanced and diverse mix of participants across demographic strata.

Potential imbalances in the collected data were addressed by utilizing the Synthetic Minority Over-sampling Technique combined with the Edited Nearest Neighbors (SMOTE-ENN) method [24]. Synthetic samples were generated using SMOTE for underrepresented age and gender groups, thereby enhancing their representation in the dataset. Data integrity was maintained by ensuring synthetic samples preserved realistic value ranges and correlational patterns consistent with the original dataset. After oversampling, the ENN technique was applied to remove noisy samples and outliers, resulting in a final balanced dataset of 12,522 samples. This dataset maintained statistical coherence with the original while achieving improved representational balance across demographic groups, ensuring robust foundations for subsequent analyses.

### 2.2. Data collection

A structured online questionnaire measuring internet access, digital literacy, and the effect of online health information on personal health was used to gather study data. For the purposes of this analysis, 34 items from the questionnaire were selected to explore key dimensions of digital health access, behavior, and perceptions across ten Arab countries. It was structured into six main sections: sociodemographic characteristics, internet access and digital divide, confidence in digital skills, usage of digital platforms for health purposes, trust in online health information sources, and the perceived impact of digital tools on health.

The sociodemographic section comprised six items, covering age, sex, country of residence, locality (urban or non-urban), education level, and employment status. The section on internet access and digital divide included three items focused on connection consistency, the extent to which internet access affected healthcare access, and agreement on limited access to health services. Five items assessed participants’ confidence in various digital health competencies, including search engines, health information sources, health applications, usage of digital information, and AI-driven applications. The questionnaire also explored eight aspects of how frequently participants used digital platforms for health-related purposes, such as social media, symptom searching, appointment booking, and participation in online health communities. Seven items evaluated trust levels in online health information sources, including government, hospitals, universities, social media, and blogs. Finally, five items captured participants’ views on the digital impact on health, specifically how digital literacy, online platforms, and information environments affect the quality and equity of health services.

To ensure content validity, the questionnaire was developed through a thorough review of current literature on digital health behavior and adapted to suit the regional context of the participating countries. Public health and digital technology experts reviewed the draft instrument to confirm the appropriateness, clarity, and relevance of the items. A pilot test involving 60 respondents from a range of countries was conducted to assess clarity and face validity. Feedback from the pilot was used to revise certain wording and improve flow. The instrument’s internal consistency was evaluated using Cronbach’s alpha, with an overall score of 0.87, indicating high reliability across the scales, particularly in the sections related to digital confidence and platform usage.

The survey was available online through the Al-Quds University platform in both Arabic and English to accommodate language preferences. It can still be accessed at http://bcite.org/di. Data collection occurred between April 1st and May 17th, 2024, to ensure a representative and varied sample was obtained.

### 2.3. Ethics statement

Ethical approval was obtained from the Al-Quds University Research Ethical Committee and the IRBs of participating countries (March 30, 2024; Ref No: 384/REC/2024). To protect privacy and confidentiality, all data were anonymized following ethical guidelines including the Declaration of Helsinki and local regulations.

### 2.4. Participants

All participants provided written informed consent before participation, acknowledging the study’s objectives to examine how internet access influences health equity, understand digital literacy’s role in health behaviors, analyze digital platforms’ effects on health decisions, and assess digital policy implications for health services in the MENA region. Participants were informed that interviews would last approximately 20 minutes and were required to sign consent forms indicating their agreement to participate, with separate consent obtained for adult participants (18 years and older). Contact information was collected for follow-up purposes. Participants were assured that their identities would not be disclosed in any resulting publications.

### 2.5. Study variables

Table 1 shows a complete list of the study variables and the categories according to which they were measured.

**Table 1:** Variables and categories for digital health information study.

Section	Variable	Categories/Description
**Sociodemographic**	Age	18-34 years, 35–44 years, 45–64 years
	Sex	Male, Female
	Country of Residence	Bahrain, Egypt, Jordan, Kuwait, Lebanon, Morocco, Palestine, Saudi Arabia, Tunisia, UAE
	Locality	Urban, Non-Urban
	Education Level	Secondary or less, Bachelor’s Degree, Graduate Degree
	Employment Status	Employed, Unemployed, Student
**Internet Access and Digital Divide**	Connection Consistency	Unreliable, Moderately Reliable, Reliable
	Affect Access to Healthcare	Low, Moderate, High
	Limited Access to Health Services	Disagree, Neutral, Agree
**Confidence in Digital Skills**	Confidence in Search Engines	Low, Medium, High
	Confidence in Health Information Sources	Low, Medium, High
	Confidence in Health Apps	Low, Medium, High
	Confidence in Using Health Information	Low, Medium, High
	Confidence in AI Applications	Low, Medium, High
**Usage of Digital Platforms for Health Purposes**	Social Media Usage	Low, Medium, High Frequency
	Health Monitoring Apps Usage	Low, Medium, High Frequency
	Online Forums Participation	Low, Medium, High Frequency
	Frequency of Searching General Health Information	Low, Medium, High Frequency
	Frequency of Looking Up Symptoms Online	Low, Medium, High Frequency
	Frequency of Following Health Influencers	Low, Medium, High Frequency
	Frequency of Participating in Online Health Groups	Low, Medium, High Frequency
	Frequency of Booking Medical Appointments Online	Low, Medium, High Frequency
**Trust in Online Health Information Sources**	Trust in Government Health Information	Low, Moderate, High
	Trust in Hospital Information	Low, Moderate, High
	Trust in Health News	Low, Moderate, High
	Trust in University Health Information	Low, Moderate, High
	Trust in Health Forums	Low, Moderate, High
	Trust in Social Media Platforms	Low, Moderate, High
	Trust in Health Blogs	Low, Moderate, High
**Digital Impact on Health**	Digital Literacy Impact	Negative, Neutral, Positive
	Digital Platforms Impact	Negative, Neutral, Positive
	Online Information Environment Impact	Negative, Neutral, Positive
	Quality of Health Services Impact	Negative, Neutral, Positive
	Equitable Access to Health Information Impact	Negative, Neutral, Positive

### 2.6. Data analysis

Data analysis was performed using IBM SPSS Statistics Version 23 and Python Version 1.6. The SPSS was used for descriptive statistics, univariate analysis, and logistic regression to explore associations between sociodemographic factors and digital health behaviors. Univariate analysis was performed using chi-square tests to examine the individual association between each independent variable and the main outcome variables related to digital health behaviors. Logistic regression modeling was then applied to assess the combined effects of multiple variables on digital health outcomes. Results are presented as odds ratios (OR) with 95% confidence intervals (CI) and significance level (P-Value).

## 3. Results

### 3.1. Sample characteristics

Table 2 presents the percentage distribution of sociodemographic characteristics across age groups and sex categories for the collected responses. The geographic distribution shows considerable diversity, with Saudi Arabia representing the largest proportion at 18.9%, followed by Kuwait at 16.5%, and Jordan at 10.5%. Egypt contributes 10% of the sample, while other Middle Eastern and North African countries including Bahrain, Lebanon, Morocco, Palestine, Tunisia, and the UAE collectively account for the remaining participants.

**Table 2:** Percentage distribution of sociodemographic variables by age group and sex.

Variables/Categories		Age group			Sex		Total
		18-34	35-44	45-64	Male	Female	
		Row Count (%)	Row Count (%)	Row Count (%)	Row Count (%)	Row Count (%)	Row Count (%)
**Country of Residence**	Bahrain	114(53.5)	39(18.3)	60(28.2)	110(51.6)	103(48.4)	213(3.4)
	Egypt	126(20.2)	157(25.1)	342(54.7)	479(76.6)	146(23.4)	625(10)
	Jordan	128(19.4)	124(18.8)	409(61.9)	145(21.9)	516(78.1)	661(10.5)
	Kuwait	439(42.4)	236(22.8)	361(34.8)	516(49.8)	520(50.2)	1036(16.5)
	Lebanon	214(45.9)	93(20)	159(34.1)	201(43.1)	265(56.9)	466(7.4)
	Morocco	297(66.4)	66(14.8)	84(18.8)	334(74.7)	113(25.3)	447(7.1)
	Palestine	319(59.6)	111(20.7)	105(19.6)	165(30.8)	370(69.2)	535(8.5)
	Saudi Arabia	442(37.3)	137(11.6)	606(51.1)	648(54.7)	537(45.3)	1185(18.9)
	Tunisia	195(40.9)	151(31.7)	131(27.5)	181(37.9)	296(62.1)	477(7.6)
	UAE	210(33.1)	153(24.1)	272(42.8)	382(60.2)	253(39.8)	635(10.1)
**Locality**	Urban	2027(37.3)	1108(20.4)	2293(42.2)	2677(49.3)	2751(50.7)	5428(86.4)
	Non-Urban	457(53.6)	159(18.7)	236(27.7)	484(56.8)	368(43.2)	852(13.6)
**Education Level**	Secondary or less	522(44.4)	145(12.3)	509(43.3)	686(58.3)	490(41.7)	1176(18.7)
	Bachelor’s Degree	1354(44.3)	590(19.3)	1115(36.4)	1295(42.3)	1764(57.7)	3059(48.7)
	Graduate Degree	608(29.7)	532(26)	905(44.3)	1180(57.7)	865(42.3)	2045(32.6)
**Employment Status**	Employed	992(29)	993(29)	1437(42)	1939(56.7)	1483(43.3)	3422(54.5)
	Unemployed	420(25.2)	202(12.1)	1043(62.6)	663(39.8)	1002(60.2)	1665(26.5)
	Student	1072(89.9)	72(6)	49(4.1)	559(46.9)	634(53.1)	1193(19)

The age distribution reveals a pronounced skew toward younger participants, as the 18–34 age group comprises nearly half of the sample at 47.3%. In contrast, the 35–44 age group represents 28.5%, and the 45–64 age group accounts for 24.2%. Gender distribution demonstrates a slight female majority at 54.3% compared to 45.7% males. Furthermore, urban residents constitute the overwhelming majority at 86.4%, while rural participants represent only 13.6% of the sample.

Educational attainment levels are notably high throughout the sample. Nearly half of participants hold bachelor’s degrees (48.7%), and an additional 32.6% possess graduate degrees. Consequently, only 18.7% have secondary education or less. Employment patterns reveal that 54.5% of participants are currently employed, while 26.5% are unemployed and 19% are students.

### 3.2. Data balancing and quality Validation

To ensure representativeness and mitigate demographic imbalance, the SMOTE-ENN algorithm was applied, expanding the dataset from 6,280–12,522 records. The augmentation achieved a uniform sex ratio and redistributed age categories. Country-level distributions before and after augmentation are summarized in Table 3, confirming consistent demographic alignment and elimination of gender bias across all participating countries.

**Table 3:** Country-level distribution among age and sex before and after SMOTE-ENN.

Country	Total Count B(A)	Age 18–34 B(A)	Age 35–44 B(A)	Age 45–64 B(A)	Male % B(A)
**Bahrain**	213(348)	95(95)	19(21)	39(116)	51.6(50.0)
**Egypt**	625(1488)	50(201)	76(295)	157(496)	76.6(50.0)
**Jordan**	661(2196)	30(182)	98(550)	124(732)	21.9(50.0)
**Kuwait**	1036(1368)	169(164)	270(292)	236(456)	49.8(50.0)
**Lebanon**	466(894)	160(214)	54(84)	93(298)	43.1(50.0)
**Morocco**	447(1308)	194(296)	103(140)	66(436)	74.7(50.0)
**Palestine**	535(1194)	155(190)	164(208)	111(398)	30.8(50.0)
**Saudi Arabia**	1185(1884)	223(327)	219(301)	137(628)	54.7(50.0)
**Tunisia**	477(624)	80(82)	115(126)	151(208)	37.9(50.0)
**UAE**	635(1218)	68(142)	142(264)	153(406)	60.2(50.0)

• **B:** Sample Count Before Augmentation.

• **A:** Sample Count After Augmentation.

Data integrity was validated through a multi-layered quality assessment. Among all variables, mean and standard-deviation shifts remained within 0.03–1.82% and 0.02–1.66%, respectively. Most of the key bivariate associations retained statistical significance after balancing (all p < 0.001), including the relationships between digital literacy and health improvement (χ² = 505.11 → 751.59) and internet access with health decision-making (χ² = 396.25 → 980.78). Odds-ratio comparisons confirmed effect-size stability (variance < 12%) for all tested predictors, ensuring that synthetic samples neither inflated nor attenuated underlying associations. Overall, these findings demonstrate that SMOTE-ENN substantially improved demographic balance and statistical power while maintaining the empirical validity and interpretive coherence of the original dataset.

### 3.3. Internet access and digital divide

Table 4 presents key indicators relating to internet access, reliability, and digital education across the surveyed countries. 93.9% of participants reported internet access, indicating a high level of overall digital connectivity in the region. However, internet reliability remained a challenge for a significant minority: 11.5% of respondents reported having unreliable connections. This issue was more evident in Morocco, Kuwait, Lebanon, and Palestine, suggesting inconsistencies in service quality that could hinder effective use of digital health tools. In contrast, participants in Jordan and the UAE reported relatively stable internet access, reflecting stronger infrastructure and digital readiness.

**Table 4:** The percentage distribution of internet access and digital divide among participating countries.

	Internet Access		Internet Consistency			Education on Internet Usage	
Country	No n (%)	Yes n (%)	Unreliable n (%)	Moderate n (%)	Reliable n (%)	No n (%)	Yes n (%)
**Bahrain**	32(9.2)	316(90.8)	37(10.6)	149(42.8)	162(46.6)	243(69.8)	105(30.2)
**Egypt**	62(4.2)	1426(95.8)	113(7.6)	744(50)	631(42.4)	532(35.8)	956(64.2)
**Jordan**	16(0.7)	2180(99.3)	129(5.9)	427(19.4)	1640(74.7)	1769(80.6)	427(19.4)
**Kuwait**	142(10.4)	1226(89.6)	273(20)	642(46.9)	453(33.1)	819(59.9)	549(40.1)
**Lebanon**	92(10.3)	802(89.7)	128(14.3)	441(49.3)	325(36.4)	685(76.6)	209(23.4)
**Morocco**	170(13)	1138(87)	283(21.6)	384(29.4)	641(49)	1247(95.3)	61(4.7)
**Palestine**	78(6.5)	1116(93.5)	148(12.4)	410(34.3)	636(53.3)	948(79.4)	246(20.6)
**Saudi Arabia**	124(6.6)	1760(93.4)	171(9.1)	939(49.8)	774(41.1)	1214(64.4)	670(35.6)
**Tunisia**	8(1.3)	616(98.7)	51(8.2)	315(50.5)	258(41.3)	560(89.7)	64(10.3)
**UAE**	34(2.8)	1184(97.2)	101(8.3)	152(12.5)	965(79.2)	924(75.9)	294(24.1)
**Total**	758(6.1)	11764(93.9)	1434(11.5)	4603(36.8)	6485(51.8)	8941(71.4)	3581(28.6)

A deeper digital divide emerged in relation to digital education. 71.4% of respondents had not received any formal instruction on internet use. This points to a critical gap in digital literacy, particularly severe in Morocco and Tunisia, where over 89% of participants reported a lack of digital education. Comparable rates in Jordan and Palestine suggest that even countries with relatively high access are not adequately addressing digital skill development.

### 3.4. Digital determinants and health outcomes

Table 5 illustrates how participants across different countries perceived the influence of digital determinants, such as digital literacy and internet access, on their health outcomes. 32.9%—approximately one-third—of respondents reported noticeable improvements in their personal health. Respondents from Egypt, Lebanon, and Saudi Arabia indicated that access to digital tools and information had enhanced their ability to manage their health more effectively, whereas participants from Morocco, Jordan, Bahrain, and Palestine were more likely to report limited perceived health benefits from digital access.

**Table 5:** The percentages of overall digital determinants impact on health among participating countries.

	Bahrain n (%)	Egypt n (%)	Jordan n (%)	Kuwait n (%)	Lebanon n (%)	Morocco n (%)	Palestine n (%)	Saudi Arabia n (%)	Tunisia n (%)	UAE n (%)	Total n (%)
**Perceived Health Improvement**											
**Low**	109(31.3)	203(13.6)	757(34.5)	374(27.3)	164(18.3)	648(49.5)	349(29.2)	352(18.7)	157(25.2)	98(8)	3211(25.6)
**Moderate**	191(54.9)	341(22.9)	907(41.3)	643(47)	377(42.2)	519(39.7)	481(40.3)	795(42.2)	286(45.8)	651(53.4)	5191(41.5)
**High**	48(13.8)	944(63.4)	532(24.2)	351(25.7)	353(39.5)	141(10.8)	364(30.5)	737(39.1)	181(29)	469(38.5)	4120(32.9)
**Ability to manage my health.**											
**Negative**	80(23)	106(7.1)	626(28.5)	445(32.5)	142(15.9)	332(25.4)	383(32.1)	537(28.5)	41(6.6)	151(12.4)	2843(22.7)
**Neutral**	188(54)	578(38.8)	507(23.1)	608(44.4)	388(43.4)	476(36.4)	292(24.5)	822(43.6)	125(20)	524(43)	4508(36)
**Positive**	80(23)	804(54)	1063(48.4)	315(23)	364(40.7)	500(38.2)	519(43.5)	525(27.9)	458(73.4)	543(44.6)	5171(41.3)
**Health behaviours and decisions**											
**Negative**	78(22.4)	79(5.3)	255(11.6)	346(25.3)	146(16.3)	334(25.5)	147(12.3)	288(15.3)	65(10.4)	59(4.8)	1797(14.4)
**Neutral**	223(64.1)	440(29.6)	1099(50)	716(52.3)	440(49.2)	591(45.2)	479(40.1)	933(49.5)	271(43.4)	519(42.6)	5711(45.6)
**Positive**	47(13.5)	969(65.1)	842(38.3)	306(22.4)	308(34.5)	383(29.3)	568(47.6)	663(35.2)	288(46.2)	640(52.5)	5014(40)
**The impact of online information on managing my health**											
**Negative**	68(19.5)	70(4.7)	277(12.6)	301(22)	131(14.7)	168(12.8)	187(15.7)	381(20.2)	54(8.7)	19(1.6)	1656(13.2)
**Neutral**	173(49.7)	595(40)	1193(54.3)	688(50.3)	463(51.8)	737(56.3)	505(42.3)	819(43.5)	290(46.5)	564(46.3)	6027(48.1)
**Positive**	107(30.7)	823(55.3)	726(33.1)	379(27.7)	300(33.6)	403(30.8)	502(42)	684(36.3)	280(44.9)	635(52.1)	4839(38.6)
**The effect of digital technology on the quality of received healthcare services**											
**Negative**	55(15.8)	98(6.6)	215(9.8)	303(22.1)	122(13.6)	113(8.6)	71(5.9)	288(15.3)	56(9)	22(1.8)	1343(10.7)
**Neutral**	206(59.2)	557(37.4)	1200(54.6)	722(52.8)	426(47.7)	819(62.6)	585(49)	934(49.6)	355(56.9)	728(59.8)	6532(52.2)
**Positive**	87(25)	833(56)	781(35.6)	343(25.1)	346(38.7)	376(28.7)	538(45.1)	662(35.1)	213(34.1)	468(38.4)	4647(37.1)
**Digital health technologies have contributed to equitable (gender, disadvantaged people…)**											
**Negative**	67(19.3)	161(10.8)	89(4.1)	275(20.1)	103(11.5)	63(4.8)	104(8.7)	200(10.6)	71(11.4)	66(5.4)	1199(9.6)
**Neutral**	179(51.4)	54(3.6)	642(29.2)	494(36.1)	259(29)	621(47.5)	265(22.2)	473(25.1)	272(43.6)	214(17.6)	3473(27.7)
**Positive**	102(29.3)	1273(85.6)	1465(66.7)	599(43.8)	532(59.5)	624(47.7)	825(69.1)	1211(64.3)	281(45)	938(77)	7850(62.7)

Regarding the broader impact on health management, 41.3% of respondents positively perceived digital technologies. Tunisia and Egypt reported notable impacts, suggesting that digital engagement is beginning to play a more active role in supporting day-to-day health-related decisions. Similarly, 40% of participants reported that access to digital health information positively influenced their health behaviors. Respondents in Egypt, the UAE, and Palestine were more likely to report such behavioral shifts, indicating that digital tools can drive both awareness and tangible changes in health practices.

Perceptions of digital technology’s impact on the quality of healthcare services were also notable. 37.1% of respondents believed that digital tools have enhanced healthcare quality, with Egypt and Palestine again showing the highest levels of agreement. This suggests that digital health is not only supporting individuals but is also being integrated into healthcare delivery systems to improve efficiency and responsiveness.

Finally, 62.7% of participants emphasized the role of digital technologies in promoting equitable access to healthcare. Egypt and the UAE were particularly strong in this regard, highlighting the role of national-level digital health initiatives in addressing disparities.

### 3.5. Digital literacy skills

Table 6 shows country-dependent confidence in digital literacy skills. 68.8% of respondents reported high confidence in using search engines, with the strongest levels observed in Tunisia (74.9%) and Egypt (74.4%). In contrast, Morocco (53.3%) and Saudi Arabia (61.5%) reported lower confidence levels, indicating disparities in basic digital search competencies.

**Table 6:** Percentage of digital literacy skills among participants distributed by country.

	Bahrain n (%)	Egypt n (%)	Jordan n (%)	Kuwait n (%)	Lebanon n (%)	Morocco n (%)	Palestine n (%)	KSA n (%)	Tunisia n (%)	UAE n (%)	Total n (%)
**Confidence in using search engines**											
**Low**	35(10.1)	305(20.5)	481(21.9)	259(18.9)	174(19.5)	326(24.9)	189(15.8)	504(26.8)	54(8.7)	264(21.7)	2591(20.7)
**Medium**	84(24.1)	63(4.2)	79(3.6)	352(25.7)	59(6.6)	163(12.5)	148(12.4)	272(14.4)	26(4.2)	68(5.6)	1314(10.5)
**High**	229(65.8)	1120(75.3)	1636(74.5)	757(55.3)	661(73.9)	819(62.6)	857(71.8)	1108(58.8)	544(87.2)	886(72.7)	8617(68.8)
**Confidence in using health information online resources**											
**Low**	31(8.9)	330(22.2)	421(19.2)	245(17.9)	131(14.7)	313(23.9)	148(12.4)	430(22.8)	67(10.7)	209(17.2)	2325(18.6)
**Medium**	72(20.7)	57(3.8)	95(4.3)	266(19.4)	66(7.4)	243(18.6)	142(11.9)	258(13.7)	69(11.1)	114(9.4)	1382(11)
**High**	245(70.4)	1101(74)	1680(76.5)	857(62.6)	697(78)	752(57.5)	904(75.7)	1196(63.5)	488(78.2)	895(73.5)	8815(70.4)
**Confidence in using health Apps**											
**Low**	90(25.9)	425(28.6)	660(30.1)	286(20.9)	227(25.4)	475(36.3)	293(24.5)	400(21.2)	124(19.9)	225(18.5)	3205(25.6)
**Medium**	113(32.5)	142(9.5)	402(18.3)	354(25.9)	99(11.1)	191(14.6)	284(23.8)	376(20)	105(16.8)	189(15.5)	2255(18)
**High**	145(41.7)	921(61.9)	1134(51.6)	728(53.2)	568(63.5)	642(49.1)	617(51.7)	1108(58.8)	395(63.3)	804(66)	7062(56.4)
**Confidence in understanding and using online health information for decision-making**											
**Low**	39(11.2)	312(21)	397(18.1)	216(15.8)	237(26.5)	460(35.2)	194(16.2)	336(17.8)	93(14.9)	148(12.2)	2432(19.4)
**Medium**	75(21.6)	76(5.1)	310(14.1)	364(26.6)	82(9.2)	203(15.5)	173(14.5)	219(11.6)	91(14.6)	125(10.3)	1718(13.7)
**High**	234(67.2)	1100(73.9)	1489(67.8)	788(57.6)	575(64.3)	645(49.3)	827(69.3)	1329(70.5)	440(70.5)	945(77.6)	8372(66.9)
**Confidence in using ChatGPT and AI Apps**											
**Low**	128(36.8)	417(28)	1053(48)	544(39.8)	231(25.8)	680(52)	448(37.5)	665(35.3)	236(37.8)	522(42.9)	4924(39.3)
**Medium**	54(15.5)	79(5.3)	232(10.6)	301(22)	128(14.3)	264(20.2)	189(15.8)	309(16.4)	102(16.3)	218(17.9)	1876(15)
**High**	166(47.7)	992(66.7)	911(41.5)	523(38.2)	535(59.8)	364(27.8)	557(46.6)	910(48.3)	286(45.8)	478(39.2)	5722(45.7)
**Confidence in the use of social media to access or share health information**											
**Low**	164(47.1)	179(12)	998(45.4)	510(37.3)	272(30.4)	704(53.8)	396(33.2)	509(27)	297(47.6)	470(38.6)	4499(35.9)
**Medium**	129(37.1)	607(40.8)	746(34)	583(42.6)	321(35.9)	359(27.4)	520(43.6)	1031(54.7)	240(38.5)	478(39.2)	5014(40)
**High**	55(15.8)	702(47.2)	452(20.6)	275(20.1)	301(33.7)	245(18.7)	278(23.3)	344(18.3)	87(13.9)	270(22.2)	3009(24)

70.4% of participants expressed high confidence in using online health information, with Lebanon (78.0%) and Tunisia (78.2%) reporting the highest rates. However, Egypt and Morocco showed greater uncertainty in this area, with 37.1% and 36.5% of respondents reporting low confidence, suggesting a need for targeted interventions to strengthen digital health literacy.

Confidence in using mobile health applications showed more variability. While 56.4% of the total sample expressed high confidence, the UAE (72.2%) and Lebanon (67.5%) stood out as the most digitally engaged in this domain. Morocco (43.7%) and Jordan (36.8%) had the highest percentages of reported low confidence, showing a need for focused educational efforts.

66.9% of respondents reported high confidence in using online health information for decision-making. The UAE (77.8%), followed by Egypt (73.2%) were leaders in this regard, whereas Morocco (47.2%) and Lebanon (41.2%) showed the lowest levels of confidence.

Confidence in using artificial intelligence tools such as ChatGPT was generally lower. Only 45.7% of participants indicated high confidence, with Egypt (59.2%) and Lebanon (52.1%) demonstrating the most familiarity and trust. Morocco (54.8%) and Jordan (48.3%) reported the highest levels of low confidence, indicating the need for broader AI literacy programs.

35.9% of participants reported low confidence in social media usage for sharing health information, suggesting that while social media is widely used, its role in reliable health communication remains limited due to concerns about misinformation or credibility.

### 3.6. Trust in sources of online health information

Table 7 explores trust in various sources of online health information. Government health websites garnered exceptionally high trust, with 42.9% of participants expressing strong confidence. The UAE (68.1%), Bahrain (60.9%), and Saudi Arabia (57.2%) reported the highest levels of trust in these official sources, while Kuwait (23.7%), Lebanon (24.4%), and Morocco (26.5%) recorded the lowest.

**Table 7:** The distribution of participants’ trust in the online health information by country.

	Bahrain n (%)	Egypt n (%)	Jordan n (%)	Kuwait n (%)	Lebanon n (%)	Morocco n (%)	Palestine n (%)	Saudi Arabia n (%)	Tunisia n (%)	AE n (%)	Total n (%)
**Government health websites**											
**Low**	85(24.4)	215(14.4)	520(23.7)	468(34.2)	300(33.6)	439(33.6)	354(29.6)	360(19.1)	173(27.7)	151(12.4)	3065(24.5)
**Moderate**	51(14.7)	686(46.1)	662(30.1)	468(34.2)	380(42.5)	504(38.5)	441(36.9)	447(23.7)	203(32.5)	238(19.5)	4080(32.6)
**High**	212(60.9)	587(39.4)	1014(46.2)	432(31.6)	214(23.9)	365(27.9)	399(33.4)	1077(57.2)	248(39.7)	829(68.1)	5377(42.9)
**Hospital or clinical websites**											
**Low**	96(27.6)	92(6.2)	474(21.6)	384(28.1)	208(23.3)	371(28.4)	322(27)	307(16.3)	177(28.4)	124(10.2)	2555(20.4)
**Moderate**	77(22.1)	996(66.9)	837(38.1)	458(33.5)	225(25.2)	464(35.5)	355(29.7)	553(29.4)	241(38.6)	412(33.8)	4618(36.9)
**High**	175(50.3)	400(26.9)	885(40.3)	526(38.5)	461(51.6)	473(36.2)	517(43.3)	1024(54.4)	206(33)	682(56)	5349(42.7)
**Health-focused news sites**											
**Low**	154(44.3)	160(10.8)	816(37.2)	401(29.3)	241(27)	391(29.9)	369(30.9)	305(16.2)	228(36.5)	403(33.1)	3468(27.7)
**Moderate**	116(33.3)	934(62.8)	920(41.9)	540(39.5)	431(48.2)	690(52.8)	446(37.4)	813(43.2)	291(46.6)	532(43.7)	5713(45.6)
**High**	78(22.4)	394(26.5)	460(20.9)	427(31.2)	222(24.8)	227(17.4)	379(31.7)	766(40.7)	105(16.8)	283(23.2)	3341(26.7)
**Universities and educational institutions websites.**											
**Low**	77(22.1)	170(11.4)	416(18.9)	419(30.6)	236(26.4)	255(19.5)	285(23.9)	393(20.9)	137(22)	170(14)	2558(20.4)
**Moderate**	88(25.3)	724(48.7)	466(21.2)	514(37.6)	305(34.1)	447(34.2)	452(37.9)	748(39.7)	164(26.3)	431(35.4)	4339(34.7)
**High**	183(52.6)	594(39.9)	1314(59.8)	435(31.8)	353(39.5)	606(46.3)	457(38.3)	743(39.4)	323(51.8)	617(50.7)	5625(44.9)
**Health forums and communities**											
**Low**	134(38.5)	177(11.9)	1213(55.2)	514(37.6)	204(22.8)	460(35.2)	484(40.5)	438(23.2)	266(42.6)	368(30.2)	4258(34)
**Moderate**	112(32.2)	613(41.2)	721(32.8)	513(37.5)	331(37)	482(36.9)	457(38.3)	859(45.6)	209(33.5)	579(47.5)	4876(38.9)
**High**	102(29.3)	698(46.9)	262(11.9)	341(24.9)	359(40.2)	366(28)	253(21.2)	587(31.2)	149(23.9)	271(22.2)	3388(27.1)
**Social media platforms**											
**Low**	153(44)	217(14.6)	1431(65.2)	509(37.2)	503(56.3)	809(61.9)	531(44.5)	692(36.7)	419(67.1)	598(49.1)	5862(46.8)
**Moderate**	106(30.5)	946(63.6)	478(21.8)	588(43)	297(33.2)	434(33.2)	483(40.5)	825(43.8)	152(24.4)	471(38.7)	4780(38.2)
**High**	89(25.6)	325(21.8)	287(13.1)	271(19.8)	94(10.5)	65(5)	180(15.1)	367(19.5)	53(8.5)	149(12.2)	1880(15)
**Blogs and personal websites**											
**Low**	184(52.9)	240(16.1)	1551(70.6)	662(48.4)	585(65.4)	957(73.2)	594(49.7)	1066(56.6)	402(64.4)	842(69.1)	7083(56.6)
**Moderate**	115(33)	893(60)	385(17.5)	495(36.2)	198(22.1)	248(19)	465(38.9)	569(30.2)	199(31.9)	331(27.2)	3898(31.1)
**High**	49(14.1)	355(23.9)	260(11.8)	211(15.4)	111(12.4)	103(7.9)	135(11.3)	249(13.2)	23(3.7)	45(3.7)	1541(12.3)

Trust in hospital and clinical websites followed a similar pattern, with 42.7% of respondents indicating high trust. The UAE (56.0%), Saudi Arabia (54.4%), and Lebanon (51.6%) ranked highest, reflecting confidence in institutional healthcare sources. Lower levels in Tunisia, Morocco, and Kuwait (each around 28%) suggest varying levels of perceived credibility or exposure to these platforms.

Health news websites received more mixed evaluations, with only 26.7% of respondents expressing high trust. Trust was strongest in Saudi Arabia (38.7%), Palestine (34.9%), and Kuwait (33.5%), which may reflect stronger engagement with health media in these countries. However, distrust was most common in Jordan (18.7%), Tunisia (19.4%), and the UAE (19.9%), indicating concerns about sensationalism or misinformation in health journalism.

Universities and educational institutions were the most trusted source overall, with 44.9% of respondents expressing high trust. This reflects a consistent belief in the credibility of academic sources. Jordan (57.2%), Bahrain (54.8%), and Tunisia (52.5%) reported the highest trust in these sources, whereas Kuwait (29.5%), Lebanon (30.2%), and Palestine (32.1%) reported lower levels, possibly due to less direct engagement with academic health content.

Health forums and online communities showed considerable variability. Egypt had the highest trust (46.9%), followed by Lebanon (40.2%) and Saudi Arabia (31.2%), indicating that in some contexts, peer support networks are viewed as valuable. However, concerns about accuracy and reliability in non-professional discussions reveal high levels of low trust in Jordan (20.7%), Palestine (21.1%), and Kuwait (22.4%).

Social media platforms were the least trusted overall, with only 15% of respondents expressing high trust. Distrust levels were highest in Tunisia (64.1%), Jordan (61.3%), and Morocco (59.7%). Blogs and personal websites also ranked low in trust. Even in the most trusting country, Egypt, only 23.9% of respondents expressed confidence in these sources. Trust was even lower in Jordan (7.1%), Morocco (7.4%), and the UAE (8.2%), emphasizing widespread skepticism toward informal or non-institutional health sources.

### 3.7. Logistic regression

Table 8 presents logistic regression analysis results, including odds ratios (OR) and confidence intervals (CI), for various digital determinants impacting health improvement in the Gulf, North Africa, and the Levant. The determinants analyzed comprised sociodemographic factors, internet usage, and digital literacy aspects, with univariate significance testing applied to identify statistical associations.

**Table 8:** Logistic regression analysis of digital determinants’ impact on health improvement across MENA regions alongside univariate significance indicators.

Determinants	Golf Region OR (95% CI)	North Africa Region OR (95% CI)	Levant Region OR (95% CI)
**Sex**	0.92(0.82–1.04) b	1.29(1.12–1.49) b**	0.81(0.71–0.93) b*
**Age**	0.9(0.83–0.96) b*	0.67(0.61–0.73) a**	0.85(0.78–0.92) a**
**Locality**	1.63(1.3–2.03) a**	0.54(0.3–0.97) b*	1.45(1.14–1.83) b*
**Education**	0.91(0.82-1.02)	2.41(1.88–3.1) a**	0.88(0.75–1.02) a
**Employment**	0.81(0.73–0.9) a**	1.04(0.88–1.23) a	0.86(0.74–0.99) a*
**Internet Connection Consistency**	1.22(1.08–1.36) a**	0.35(0.28–0.44) a**	0.95(0.82–1.11) a
**Affect** **Access to Healthcare**	1.66(1.5–1.83) a**	2.29(1.9–2.77) a**	1.68(1.5–1.87) a**
**Digital Literacy**	1.17(1.01–1.35) a*	4.17(2.9–6) a**	2.28(1.86–2.79) a**
**Distinguish between Information**	1.53(1.37–1.7) a**	1.19(0.93-1.52)	0.41(0.35–0.49) a**
**Use internet to make decision**	1.74(1.57–1.93) a**	2.19(1.74–2.77) a**	1.81(1.56–2.09) a**
**Search Engine**	0.85(0.77–0.93) a**	1.01(0.79-1.29)	0.71(0.62–0.8) a**
**Healthsource**	0.91(0.83-1.01) **	0.72(0.56–0.92) b*	1.67(1.45–1.93) a**
**Healthcare Apps**	1.1(1-1.21) *	1.91(1.6–2.29) a**	0.81(0.72–0.92) a**
**Using health information**	1(0.9-1.11)	2.02(1.62–2.51) a**	1.11(0.98-1.27)
**Use AI Apps**	1.15(1.06–1.25) b**	1.17(0.98-1.39)	1.71(1.55–1.9) a**
**Social Media**	0.95(0.85-1.06)	0.63(0.49–0.82) a**	0.34(0.29–0.4) a**
**Health Monitoring**	1.29(1.16–1.43) a**	0.81(0.63–1.04) a	1.11(0.97-1.26)
**Online Forms**	1.1(0.98–1.24) b	1.05(0.8–1.4) b	1.81(1.56–2.11) a*
**Search for General health**	1.21(1.07–1.36) a*	1.61(1.2–2.17) a**	1.07(0.9-1.27)
**Look up symptoms**	0.55(0.49–0.62) a**	0.63(0.47–0.83) a**	1.76(1.5–2.07) a**
**Following Influencers have healthcare content**	0.82(0.74–0.91) a**	2.24(1.79–2.79) a**	1.7(1.46–1.99) a**
**Participating in health groups**	1.42(1.27–1.59) a**	1.53(1.13-2.08) *	0.83(0.71–0.97) b*
**Booking Appointment**	1.29(1.17–1.42) a**	0.86(0.69–1.08) b	1.03(0.91-1.17)
**Government Health Information**	0.91(0.81–1.01) b	4.43(3.37–5.84) a**	1.61(1.36-1.91) **
**Hospital Information**	0.99(0.88–1.11) a	0.86(0.65–1.15) a	1.12(0.96-1.31)
**Health News**	0.92(0.82–1.03) b	2.84(2.11-3.81) **	0.81(0.7-0.94) *
**University Health information**	1.05(0.95-1.17)	1.13(0.86-1.48)	1(0.85-1.18)
**Health Forums**	0.88(0.79–0.98) b*	0.56(0.43–0.73) a**	0.99(0.85-1.14)
**Social Media Plat**	1.09(0.98-1.23)	1.25(0.93-1.68)	1.13(0.95-1.33)
**Blogs**	1.36(1.21–1.52) a**	1.74(1.32–2.3) a**	0.95(0.81-1.11)

• ***Univariate Significance***: *(a)* P ≤ 0.001; *(b)* *0.001 <* P ≤ 0.05; Else not significant.

• ***Regression Significance***: (**) P ≤ 0.001; (*) *0.001 <* P ≤ 0.05; Else not significant.

In the Gulf region, younger individuals (OR=0.9) were more likely to experience health improvements through digital means. Urban locality (OR=1.63) and access to healthcare (OR=1.66) showed strong positive effects, while unemployment reduced benefits (OR=0.81). Consistent internet connection (OR=1.22), digital literacy (OR=1.17), using the internet for decision-making (OR=1.74), AI apps (OR=1.15), and health monitoring (OR=1.29) all significantly enhanced outcomes.

In North Africa, females (OR=1.29) and younger individuals benefited more from digital health. Education (OR=2.41), access to healthcare (OR=2.29), digital literacy (OR=4.17), internet use for decision-making (OR=2.19), and health news (OR=2.84) showed strong positive effects. In contrast, rural locality (OR=0.54), inconsistent internet connection (OR=0.35), and social media use (OR=0.63) had a highly significant negative impact, showing reduced benefits.

In the Levant, males (OR=0.81) and younger individuals (OR=0.85) were more likely to experience digital health improvement. Urban locality (OR=1.45), access to healthcare (OR=1.68), digital literacy (OR=2.28), and using the internet for decision-making (OR=1.81), AI apps (OR=1.71), and health sources (OR=1.67) had positive effects. In contrast, unemployment (OR=0.86), search engines (OR=0.71), social media (OR=0.34), and health news (OR=0.8) had negative effects.

## 4. Discussion

The study explored how digital determinants of health, especially the digital divide, influence health improvements across the Arab world.

### 4.1. Role of digital literacy and internet reliability

The study identified significant disparities in digital literacy across the Arab world, which are critical determinants of health outcomes. Notable gaps in Morocco and Tunisia, stemming from variations in educational policies, socioeconomic factors, and the efficacy of digital initiatives, contrast sharply with the more integrated approached and better health outcomes seen in nations like the UAE [25]. These findings showed the necessity of strategic investments in both digital infrastructure and focused educational reforms.

The findings demonstrate that digital literacy acts as the essential bridge between technological access and tangible health outcomes. This is evident in countries with higher digital proficiency, such as Egypt, Tunisia, Lebanon, and Saudi Arabia, where participants reported more effective health self-management. The critical factor was not just the availability of digital platforms but the ability to confidently access, evaluate, and use online health information to make informed decisions. This confirms previous studies showing that effective use of digital health resources improves health management and decision-making skills [22].

Furthermore, the study highlights the role of digital literacy in leveraging advanced health systems. In Egypt and Palestine, digital health tools like electronic medical records and telemedicine improved healthcare quality by facilitating provider connection and reducing errors. However, their efficacy remained contingent upon user confidence and trust in the systems [26]. This aligns with World Health Organization advocacy for national strategies to enhance digital health literacy, particularly in low- and middle-income countries, to improve health outcomes and reduce disparities [21].

This was especially clear in countries like Morocco, Jordan, Bahrain, and Palestine, where we saw lower levels of reported health improvements. In many cases, this was tied to lower digital literacy and unreliable internet access. People in these countries may have the devices, but they struggle to access or benefit from online health resources. This highlights the need for more focused efforts on digital literacy training and improving internet infrastructure. Just giving people access to technology isn’t enough, they need the knowledge and support to use it effectively [27,28].

By contrast, participants in Tunisia and Egypt reported that digital tools had a noticeable impact on how they managed their health. With more reliable access to trustworthy online information, they were better able to make healthy choices and stay informed. In these cases, it wasn’t just the availability of digital platforms that made the difference, it was people’s ability to understand and use them well. This shows that digital literacy acts as a bridge between having access and actually seeing results in personal health.

In contrast, the success of integrated national digital literacy programs in countries like the UAE illustrates a path forward. These findings collectively highlight the necessity for focused educational reforms and strategic investments in digital infrastructure. Moving beyond isolated initiatives to comprehensive, policy-driven approaches is imperative to ensure that digital health tools fulfill their potential for improving health equity across the region.

### 4.2. Trust and quality of online health information

Our findings indicated that digital health technologies are a potent but uneven force in shaping healthcare equity and management across the MENA region. The analysis reveals that their impact is profoundly mediated by two core digital determinants: reliable infrastructure and individual digital literacy.

A significant majority of participants recognized the potential of digital health to bridge geographical barriers and promote more equitable healthcare access, with the most positive impacts reported in Egypt and the UAE [29,30]. This aligns with existing literature confirming that well-implemented telehealth and health applications enhance healthcare delivery and patient outcomes [27,30–32]. However, this potential is not being uniformly realized. The study identified a clear divergence, where negative impacts were more prevalent in countries with inconsistent internet access and lower digital literacy levels. This disparity risks creating a new form of digital exclusion that can exacerbate existing health inequalities, a concern strongly echoed in prior research [33–35]. Consequently, our findings emphasize that targeted interventions to improve digital literacy and internet reliability are not merely beneficial but are a critical prerequisite for achieving equitable health outcomes across the region.

The efficacy of digital health tools is fundamentally dependent on an individual’s ability to use them. Our results confirm that digital literacy is a key empowering factor, enabling effective health management and informed decision-making [14,36,37]. This is supported by interventions demonstrating that improved digital health literacy enhances self-management behaviors and leads to better health outcomes [4,20,21]. This relationship was particularly evident in countries like Bahrain, Egypt, Jordan, and Lebanon, where digital determinants significantly influenced health improvement. For instance, higher eHealth literacy, as noted in other studies, correlates strongly with better access to online health resources and positive health decisions [38,39].

Furthermore, the utility of digital health information is contingent upon trust. We found that trust in online health sources varied widely, with higher credibility assigned to official channels like government and hospital websites, while blogs and social media were viewed with greater skepticism, particularly in Jordan and Morocco. This finding is critical, as it aligns with the established principle that trust in credible online information is crucial for effective patient engagement and the successful adoption of digital health tools [3,14,20,31].

Finally, logistic regression analysis highlighted significant regional variations in the impact of digital determinants between the Gulf, North Africa, and the Levant. This shows the association between sociodemographic factors, digital literacy, and internet usage, suggesting that a one-size-fits-all approach is inadequate. In conclusion, while digital health offers a promising path toward more equitable and effective healthcare, its benefits are conditional. Maximizing its positive impact requires a dual focus: developing inclusive digital health policies that address infrastructure gaps and implementing educational initiatives that build population-wide digital literacy and critical appraisal skills.

### 4.3. Regional differences in digital determinants

The analysis reveals significant regional disparities in the determinants of digital health adoption, indicating the profound influence of broader socio-economic, infrastructural, and cultural contexts.

A central theme emerging across all regions is the critical importance of digital literacy and education. This factor is most pronounced in North Africa, where it demonstrates an extraordinarily high impact [40], and in the Levant, where it is closely linked to the effective use of AI applications [41]. This universal significance reinforces that technological access alone is insufficient; targeted educational programs are a prerequisite for effective digital health deployment.

Demographic and geographic factors also play a pivotal but varied role. Younger, urban populations in both the Gulf and Levant regions show significantly higher engagement, benefiting from greater digital literacy and technology adoption rates [42]. This urban-rural divide is particularly stark in North Africa, where locality has a significant negative impact, highlighting acute disparities in health technology access [43]. Furthermore, the contrasting impact of sex between regions is highly revealing. While women in North Africa, who often serve as primary caregivers, are more likely to seek digital health resources [44,45], the significant negative impact of sex in the Levant suggests that cultural and social dynamics uniquely constrain female engagement there, demanding region-specific strategies.

The data also points to significant behavioral and resource barriers. In the Gulf, employment status imposes a significant negative impact, suggesting that time constraints and work-life balance limit employed individuals’ ability to engage with digital health tools [46]. Similarly, in the Levant, social media use shows a negative correlation, potentially pointing to issues of misinformation or ineffective health communication strategies prevalent on these platforms [47].

In conclusion, these findings move beyond simple description to highlight fundamental requirements for successful digital health integration. These include reliable infrastructure, which is clearly demonstrated by the Gulf’s advantage stemming from its critical consistent internet connection [48]; comprehensive digital literacy initiatives that are urgently needed in regions like North Africa and the Levant; and culturally nuanced communication strategies, essential for addressing the unique barriers present in the Levant and rural areas.

The Levant region’s complex socio-political environment necessitates nuanced approaches to digital health deployment.

### 4.4. Policy and research implications

These findings emphasize the necessity of tailored digital health strategies. The Gulf region emphasizes consistent internet access and urban-centric initiatives that align with the Digital Health Strategy 2020–2025, promoting infrastructure development for health technology [49]. North Africa could bridge the health technology gap by enhancing digital literacy and education. Evidence shows that educational interventions significantly improve health outcomes [50]. The Levant region’s focus on AI and digital literacy suggests integrating advanced technologies with culturally sensitive health communication strategies, a recommendation supported by the World Bank’s digital health guidelines [51].

While our findings show a connection between internet use, digital confidence, and improved health outcomes, it’s important to understand that these factors don’t automatically lead to better health. They act more like steppingstones that help people access and use digital health tools. However, just being online or feeling comfortable with technology isn’t enough on its own. People also need the skills to tell the difference between reliable and unreliable information, and they need access to healthcare services where they can apply what they’ve learned. For instance, someone might spend a lot of time online but rely on inaccurate sources, which could lead to poor health decisions. On the other hand, even limited internet use can be helpful if people know where to find trustworthy information and how to use it. So, the impact of internet use and digital confidence on health should be seen as part of a bigger picture, where outcomes depend on how these tools are used and supported.

## 5. Conclusion

This study highlights how digital determinants, such as internet access, connection reliability, digital literacy, and trust in online health information, shape individuals’ ability to engage with digital health tools across the Arab region. Countries like Tunisia, Jordan, and the UAE, where digital infrastructure is stronger and digital skills are more developed, showed more favorable patterns in self-reported health-related behaviors. While digital literacy supports better use of online health resources, it does not automatically translate into higher health literacy or improved personal health. Health outcomes are influenced by a broader set of factors, including the quality of healthcare services, socioeconomic conditions, and the credibility of information sources. Simply being digitally literate does not guarantee that individuals will understand, trust, or act on health information in ways that lead to better outcomes.

Moreover, the study relied on self-reported data rather than clinical health assessments or standardized health literacy scales. Future research should incorporate more objective measures to better understand the link between digital engagement and actual health outcomes.

To move forward, digital health strategies should be tailored to local contexts, recognizing differences in infrastructure, education, and health system capacity. Investing in both digital and health literacy, improving internet reliability, and promoting trust in credible health information are essential steps toward making digital health tools more effective and equitable. These efforts are especially critical in low- and middle-income settings, where digital health holds great promise but requires thoughtful, inclusive implementation.

## 6. Study strengths and limitations

This study highlights digital determinants and health outcomes across MENA countries, leveraging geographical diversity for comparative analysis. It highlights the relevance of digital technology trends and policy implications for enhancing digital literacy and internet access to improve health outcomes and reduce disparities. However, limitations include its cross-sectional design, potential bias from self-reported data, insufficient socioeconomic detail affecting understanding of regional disparities, and insufficient measures of actual levels of health literacy, digital competence, or health status. While diverse country coverage enriches insights, findings may not be universally generalized. Variability in digital infrastructure and rapid technological changes may impact consistency and timeliness. Future research should employ longitudinal designs and more comprehensive data to address these limitations and enhance applicability.

## Supporting information

S1 Fig Graphical abstract. (PNG)
