# Supplementary figures and images for: Digital health determinants & divide in the Arab world: A cross-sectional study
Source: PLoS One. 2025 Dec 31;20(12):e0338299. doi: 10.1371/journal.pone.0338299 (PMC12755751; doi:10.1371/journal.pone.0338299)

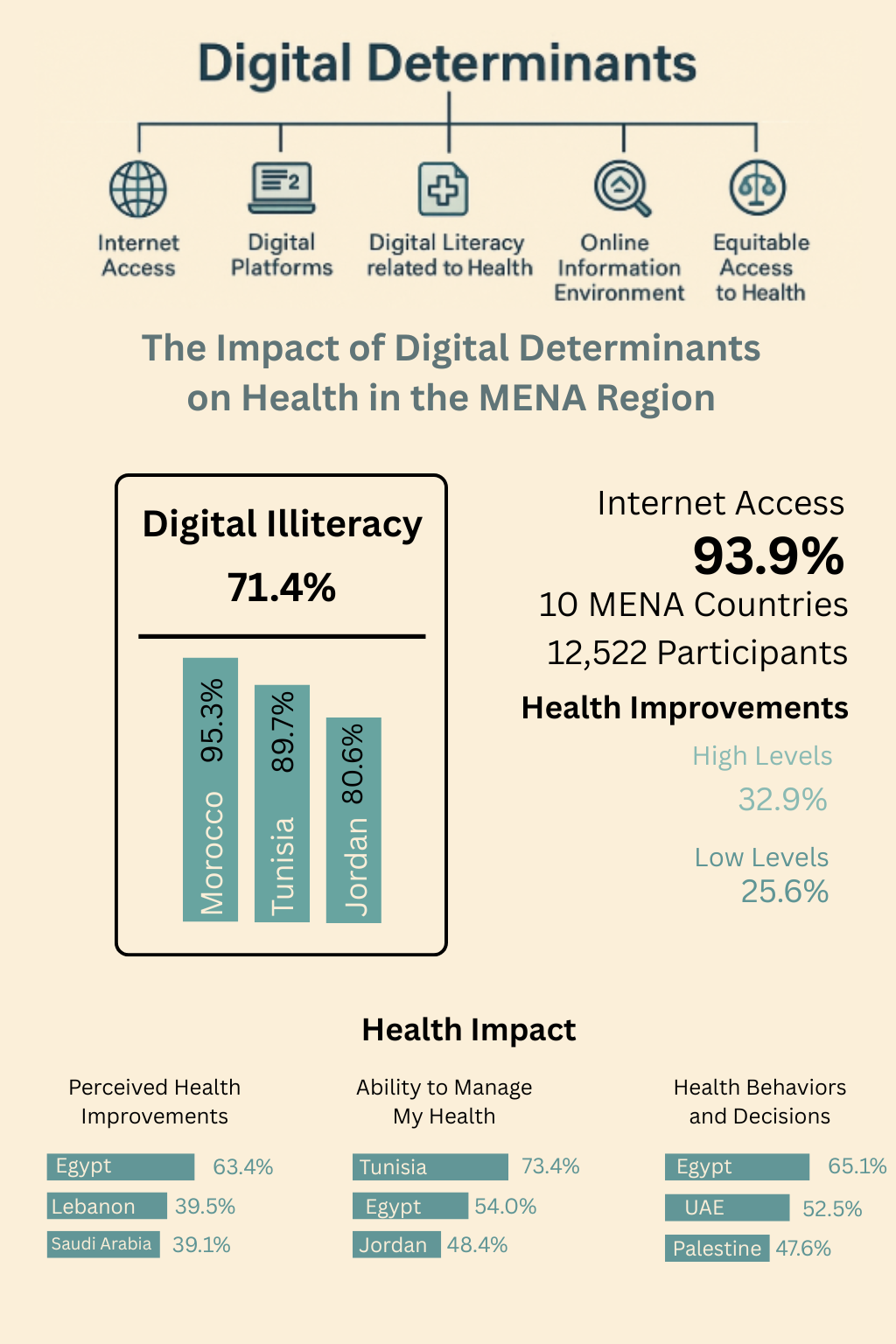

Supplement: S1 Fig — (PNG) [file pone.0338299.s001.png]
